# Editorial: The Prevalence of MDR Non-Fermenting Gram Negative Bacteria and Their Chemotherapy

**DOI:** 10.3389/fmicb.2021.664336

**Published:** 2021-07-28

**Authors:** Moutong Chen, Dongsheng Zhou, Guangchao Xu, Zhenbo Xu

**Affiliations:** ^1^Guangdong Provincial Key Laboratory of Microbial Culture Collection and Application, State Key Laboratory of Applied Microbiology Southern China, Guangdong Institute of Microbiology, Guangdong Academy of Sciences, Guangzhou, China; ^2^Beijing Institute of Microbiology and Epidemiology, Beijing, China; ^3^State Key Laboratory of Pathogen and Biosecurity of China, Beijing, China; ^4^Department of Plastic Surgery, Affiliated Hospital of Zunyi Medical University, Zunyi, China; ^5^Collaborative Innovation Center of Tissue Injury Repair and Regenerative Medicine Co-Sponsored by Province and Ministry, Zunyi Medical University, Zunyi, China; ^6^School of Food Science and Engineering, Guangdong Province Key Laboratory for Green Processing of Natural Products and Product Safety, South China University of Technology, Guangzhou, China; ^7^Department of Civil and Environmental Engineering, University of Maryland, College Park, MD, United States

**Keywords:** MDR, *Acinetobacter baumannii*, *Pseudomonas aeruginosa*, antimicrobial thermotherapy, molecular epidemiology

This Research Topic, entitled “The Prevalence of MDR Non-Fermenting Gram-Negative Bacteria and Their Chemotherapy,” has published four relevant manuscripts. The background of this topic concerns antimicrobial resistance, which is considered to be a leading concern in public health, and the high prevalence of non-fermenting Gram-negative bacteria in clinical settings has been reported from different areas all over the world. In particular, *Acinetobacter baumannii* and *Pseudomonas aeruginosa* have been often reported to cause a variety of infections types including bloodstream infection, respiratory infection, urinary infection, and wound infection. Due to the presence of multiple antibiotic resistance mechanisms, these bacterial isolates often exhibit MDR and antimicrobial treatment for their infections is greatly hampered. To control these infections, sufficient investigations into the molecular epidemiology, bacterial virulence, resistance mechanism, and antimicrobial chemotherapy strategies for these bacteria are required.

A few important and critical questions remain unclear. Firstly, what are the key environmental and bacterial factors in non-fermenting Gram-negative bacteria for survival, transmission, and infection in clinical settings? Epidemiological techniques, genetic and genomic investigations, and animal models may be used for characterization. Secondly, what are the key mechanisms for the evolution of multidrug resistance in these bacteria? The genomic basis of MDR strains should be investigated and potential interactions of these factors should be characterized. Thirdly, how do we treat the infections caused by MDR non-fermenting Gram-negative bacteria? Detailed clinical management and antimicrobial strategies should be recorded to evaluate the efficacy. As a consequence, we had proposed the current Research Topic, and we have aimed to accept manuscripts with the following scope: epidemiology of MDR non-fermenting Gram-negative bacteria in different clinical scenarios, resistance mechanism for non-fermenting Gram-negative bacteria with clear genetic evidence, and treatment of infection caused by MDR non-fermenting Gram-negative bacteria.

For the manuscripts published in this Research Topic, two were about *P. aeruginosa*, one was about both *P. aeruginosa* and *A. baumannii*, and one was about *Shigella*. As the bacterial species was concerned, the manuscripts published in this Research Topic were in accordance with our expectations and scope. In detail, two manuscripts were about studies on foodborne *P aeruginosa* with isolation from drinking water or raw milk. One manuscript, entitled “Prevalence, Virulence, Antimicrobial Resistance, and Molecular Characterization of *Pseudomonas aeruginosa* Isolates From Drinking Water in China” Wei et al., has described the prevalence, virulence, antimicrobial resistance, and molecular genotypes (sequence types) of a large scale of strains (314 strains) from 23 cities in China with a study duration of 1 year (2013–2014). A few important findings from this manuscript are as follows. Firstly, ~50% of drinking water factories were found to be positive for this pathogen. Secondly, a high occurrence of virulence genes was found among this pathogen, with >80% for all tested virulence genes except for *ExoU*. Thirdly, surprisingly and remarkably, none of the *P. aeruginosa* was found to be resistant to any single one of the 14 tested antibiotics, which may shed light on where the antimicrobial resistance has evolved in this pathogen which has been considered to be a “Super Bug.” Fourthly, the authors had also provided the sequence types of the tested strains. As far as is known, this is the first report that has provided comprehensively current knowledge on *P. aeruginosa* from drinking water (a major isolation source for this pathogen) in China, which may significantly aid in further control of food contamination by *P. aeruginosa*. One potentially interesting question from this study is the relationship between such environmental and clinical *P. aeruginosa* strains.

Another manuscript, entitled “Antibiotic Resistance Patterns of *Pseudomonas* spp. Isolated From Raw Milk Revealed by Whole Genome Sequencing” Meng et al., has described a study on *P. aeruginosa* isolated from raw milk. Firstly, the authors had performed antimicrobial resistance on 15 antimicrobial agents, then after selection, 44 strains were further subjected to genome sequencing. This manuscript has provided comprehensive knowledge on the genome of *P. aeruginosa* isolates from raw milk. However, how the genomes of these selected isolates correlate with antimicrobial resistance requires further investigation and interpretation.

The third manuscript, entitled “Antimicrobial Activity of Colistin Against Contemporary (2015–2017) *P. aeruginosa* and *A. baumannii* Isolates From a Chinese Surveillance Program” Zhang et al., had described overall MDR and carbapenem resistance (CR) rates on a very large scale of *P. aeruginosa* and *A. baumannii* with hospital origins. As reported by these authors, high MDR and CR rates were found for both pathogens during 2015–2017. Also, strains isolated from respiratory tract infection, intra-abdominal infections, and urinary tract infections were found to maintain high susceptibility rates only to colistin in the study period. However, the reason for the unique resistance to colistin requires further study.

One last manuscript, entitled “Prevalence of Plasmid-Mediated Determinants With Decreased Susceptibility to Azithromycin Among *Shigella* Isolates in Anhui, China” Liu et al. has described the azithromycin (AZM) susceptibility patterns among 517 non-duplicate *Shigella* clinical isolates (449 *S. flexneri* and 68 *S. sonnei*) in Anhui, China. The authors firstly conducted antimicrobial resistance tests, and this was followed by screening of plasmid-mediated genes by PCR and sequencing. Also, conjugation and pulsed-field gel electrophoresis were further performed. As described by these authors, increased resistance to AZM in *Shigella* isolates in the study was common, and the plasmid-mediated *mphA* gene (macrolide resistance gene) was the most frequently detected. However, concerns about the genetic context surrounding *mphA*, as well as further analysis on the evolution or acquisition of this gene, require further study.

As concluded, the above manuscripts published in this Research Topic had provided comprehensive knowledge and insight in understanding the prevalence of MDR in non-lactose fermenting Gram-negative bacteria, which may aid in further chemotherapy on such pathogens. However, limitations of this Research Topic include the relatively small number of accepted manuscripts (six submitted and four accepted) and lack of clinical studies among the accepted manuscripts despite this being one of the aims of the topic.

## Author Contributions

ZX: wrote the editorial. All authors contributed to the article and approved the submitted version.

## Conflict of Interest

The authors declare that the research was conducted in the absence of any commercial or financial relationships that could be construed as a potential conflict of interest.

## Publisher's Note

All claims expressed in this article are solely those of the authors and do not necessarily represent those of their affiliated organizations, or those of the publisher, the editors and the reviewers. Any product that may be evaluated in this article, or claim that may be made by its manufacturer, is not guaranteed or endorsed by the publisher.

